# Effect of Different Rheological Models on the Distress Prediction of Composite Pavement

**DOI:** 10.3390/ma13010229

**Published:** 2020-01-04

**Authors:** Ki Hoon Moon, Augusto Cannone Falchetto, Hae Won Park, Di Wang

**Affiliations:** 1 Korea Expressway Corporation, Pavement Research Division, 208-96, Dongbu-daro 922 beon-gil, Dongtan-myeon, Hwaseong-si 04717, Korea; moonx113@umn.edu; 2 Department of Civil & Environmental Engineering, University of Alaska Fairbanks, 1760 Tanana Loop, Fairbanks, AK 99775, USA; 3 Department of Civil Engineering, Inha University 100 Inha-ro, Michuhol-gu, Incheon 22212, Korea; czess@naver.com; 4 Department of Civil Engineering and Environmental Science, Technische Universität Braunschweig, Beethovenstraße 51 b, 38106 Braunschweig, Germany; di.wang@tu-braunschweig.de

**Keywords:** composite pavement, dynamic modulus, mechanistic-empirical pavement design guide (MEPDG), reflective cracking

## Abstract

In this paper, three different rheological models including a newly developed formulation based on the current Christensen Anderson and Marateanu (CAM) model, named sigmoidal CAM model (SCM), are used to estimate the evolution of roughness, rutting, and reflective cracking in a typical composite pavement structure currently widely adopted in South Korea. Three different asphalt mixtures were prepared and dynamic modulus tests were performed. Then, the mechanistic-empirical pavement design guide (MEPDG) was used for predicting the progression of the pavement distress and to estimate the effect of the three different models on such phenomena. It is found that the three different mathematical models provide lower and upper limits for roughness, rutting, and reflective cracking. While the CAM model may not be entirely reliable due to its inability in fitting the data in the high-temperature domain, SCM might result in moderately more conservative pavement design.

## 1. Introduction

Asphalt pavement overlay is a common practice for rehabilitating the existing concrete pavement having more than 15 to 20 years in the expressway network in South Korea [1,2]. As an alternative solution, the construction of a new concrete layer (slab) on the existing concrete pavement surface treated with a rubblization process was initially considered but finally discarded [1,2,3,4]. Two major reasons are associated with the decision of applying an overlay of asphalt material on the currently aged concrete pavement. First, re-paving concrete pavement after the rubblization process is very time demanding and costly: during the remodeling phase, the lane under construction is entirely closed off to vehicles creating consistent traffic disruption and complex management of the infrastructure for owners and contractors [1,2,3]. Second, the quality of asphalt pavements in South Korea has seen a remarkable improvement since the early 2000s. Stone mastic asphalt (SMA) mixture production and construction techniques were adopted from Germany tailoring specification and construction standards to the conditions in South Korea (e.g., asphalt binder quality improvement, aggregate gradation, plant management, and construction solutions) [2,4,5,6,7,8,9,10].

The newly developed SMA technology provides not only good mechanical performance but also high riding quality confirmed by a survey in which most of the road users affirm to prefer SMA paved road to conventional hot mix asphalt (HMA) mixture and concrete expressway [2,4,5,6,8]. Therefore, SMA overlay is preferred in South Korea for aged concrete pavement reconstruction/renovation projects with a projection in this direction for the next decade [1,2]. Moreover, a modified and enhanced mixture partly derived from the concept of SMA was developed in 2006 with the objective of further improving driving quality and safety. This novel material known as low-noise porous asphalt (LNPA) is planned to be applied in the current reconstruction projects of aged concrete pavement along within the new highway construction project Seoul-Saejong which is part of the next generation of expressway [11,12]. It should be remarked that the characteristics of LNPA used in South Korea are quite different from other porous mixtures [6,11,12]. The air void content is between 18% and 22% and only a high-grade asphalt binder with a performance grade PG 82-34 [13] is used. In addition, a high-quality aggregate with strict control at the batch plant is required. Along with conventional HMA and SMA, LNPA will find large applications in overlay layers on current aged concrete pavement in South Korea [11,12].

It should be mentioned that a more accurate approach to the evaluation of pavement distresses is needed when asphalt overlay is performed on the concrete pavement layer; this is because the structure of composite pavements is different from conventional asphalt pavements in the case of traffic load distribution [14,15]. Conventional distresses considered in asphalt pavement include rutting, fatigue cracking, thermal cracking, and potholes. However, after applying an overlay of asphalt material, the above series of distresses became less important compared to reflective cracking (RC) [14,15,16,17,18,19,20,21,22].

RC is a peculiar pavement distress mainly occurring in composite pavements. RC presents similar crack initiation and progress mechanism to the bottom-up (BU) cracking while finding a location in correspondence of the concrete pavement joints underneath the surface [14,15,16,17,18,19,20,21,22]. As a consequence of RC, the pavement surface exhibits a series of transverse cracking (similar to low temperature thermal cracking) which crosses the entire pavement transverse profile [14,15,19,22]. In order to substantially mitigate RC, optimized overlay asphalt pavement thickness (e.g., more than 8 cm) was recommended in different studies [14,15]. Nevertheless, a number of research efforts highlighted the negative impact of RC on the performance and durability of composite pavement [14,15,16,17,18,19,20,21,22]. Two major negative effects can be identified: first, a poor driving experience due to the regular presence of the cracks where RC is observed and second, serious pavement deterioration can occur not only in the layer consisting of asphalt material but also in the concrete layer due to infiltration of water and moisture. This phenomenon can reach an even more severe level compared to low temperature thermal cracking as, in the worst case, RC can develop at every location of the joints in the concrete pavement [14,15,16,17,18,19,20,21,22]. For instance, concrete pavement joints are placed every six meters in South Korea; therefore, a total of 166 RC events might be experienced every kilometer of the expressway in the worst case.

Several researchers focused on evaluating RC while proposing different solutions for mitigating such distress. Maurer et al. investigated the use of geotechnical fabrics and fibers to mitigate the effects of RC [16]. For this purpose, a specific testing site was constructed while the performance against RC was monitored in two stages after 26 and 44 months. It was found that in the short-term, the use of fabrics and fibers was providing better performance. However, in the case of the long-term response, none of the proposed solutions were proven to be cost-effective which suggests that developing durable asphalt pavement materials rather than interlayer solutions might be more efficient in mitigating RC.

Bennert et al. [18] performed field and laboratory forensic analyses of RCs to develop a framework to assess the compatibility between various types of asphalt material overlay and reflective crack-relief interlayer. Pasquini et al. [21] and Walubita et al. [23] evaluated the effect of interlayer grid reinforcements such as geo-composites on mitigating RCs by means of field sections and the use of two different HMAs. The grid reinforced section presented better performance with respect to RCs and pavement layer bonding compared to the control section; however, similar resistance to rutting along with inferior pavement smoothness was observed. A simple performance test (SPT) was developed by Zhou et al. [18] and Noory et al. [24] for measuring and evaluating the shear bond between asphalt and sub-layer to further verify the advantages (i.e., mitigate RCs) and drawbacks (i.e., reduction in bonding effect) of combining geosynthetics and different types of asphalt mixtures in the construction composite pavement. Together with experimental and field evaluations, RC was also addressed in several studies based on numerical analysis including various types of loading and concrete pavement conditions. Based on FEM simulation, crack initiation, rutting, and stress concentration mechanisms could be observed and analyzed [14,15,17,20,22].

In spite of the significant research attention devoted to RC, not many studies considered the incorporation of the mechanical properties (i.e., rheological characteristics) of asphalt mixtures such as the dynamic modulus within a global evaluation of the pavement performance taking into account aspect such as the international roughness index (IRI), rutting, top-down cracking, and RC during its expected service life. It is well known that the dynamic modulus [25] of asphalt mixtures can provide critical information on the material response [26,27,28]. Depending on the selected mathematical models used for generating master curves, experimental results of dynamic modulus can provide different predictions ultimately highly affecting the evaluation of the pavement performance with respect to different distresses such as RCs [14,15,26,27,28].

## 2. Objective and Research Approach

**Figure 1:**
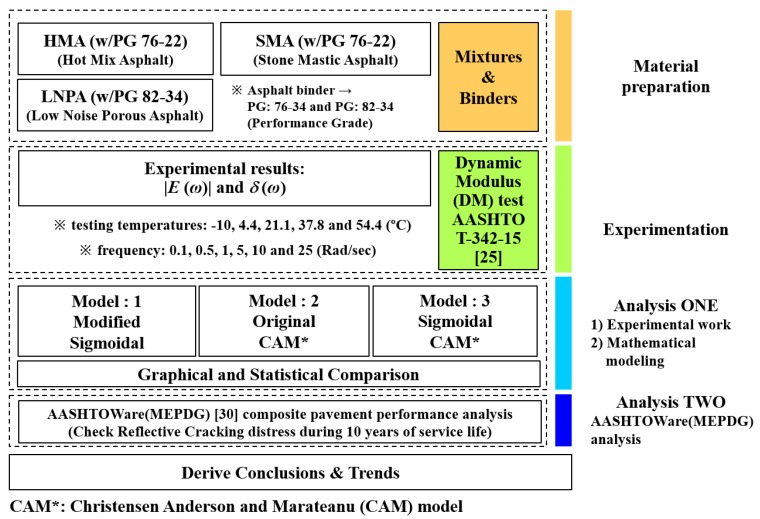
Schematic of the research approach.

In this paper, the impact of different mathematical models used for generating master curves of dynamic modulus, on the predicted performance of rehabilitated concrete pavements subjected to asphalt overlay is investigated. First, three different asphalt mixtures widely used in South Korea such as HMA, SMA, and LNPA [29] are prepared and the dynamic modulus is experimentally measured [25]. Then, three different mathematical models, including a newly developed model, are selected to analyze the rheological properties of the tested asphalt mixtures. Finally, a commercial pavement analysis software, AASHTOWare-Pavement [30], is used to evaluate the effect of different asphalt materials and mathematical modeling on the performance prediction of composite pavement, including RC resistance. The research approach adopted in the present study is shown in Figure 1.

## 3. Experimentation

A set of three asphalt mixtures consisting of a hot mix asphalt (HMA) mixture, a stone mastic asphalt (SMA) mixture, and a low noise porous asphalt (LNPA) mixture commonly applied for overlay on aged concrete pavement in South Korea are selected for this study [30]. Two different types of asphalt binder having PG 76-22 (for HMA and SMA) and PG 82-34 (for LNPA) were used for the mix design [13,31]. Schematic information on the prepared materials is presented in Table 1.

**Figure 2:**
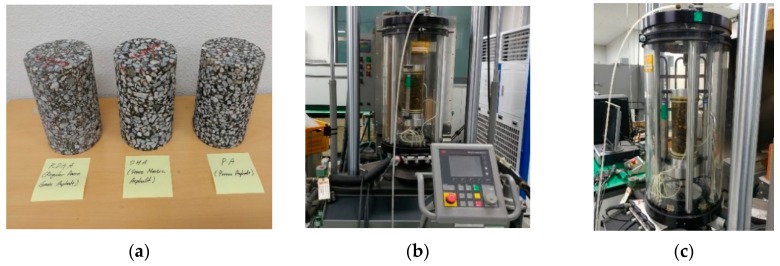
Specimens (**a**) and dynamic modulus testing setup (**b**,**c**).

Aggregate fillers and asphalt binders are obtained in a quarry and in an asphalt plant located near northern Kyeong-Gi do, Dong-Tan myeon, South Korea, respectively. The target values of air voids (AV), voids in the mineral aggregates (VMA), voids filled with asphalt (VFA) and optimum asphalt content (OAC) are set based on the current Korean and on Superpave specifications [13,31]. All asphalt mixtures are prepared in the Pavement Research Division laboratory of Korea Expressway Corporation (KECPRD) (Hwaseong, South Korea). The dynamic modulus test (DMT) is performed based on the current AASHTO T342-15 standard [25]. Three replicates per each mixture set are prepared and tested. Therefore, a total of nine asphalt mixture samples are used. Detailed experimental information of DMT is provided in Figure 2 and Table 2, respectively.

The dynamic modulus, |E*(*ω*)| and corresponding phase angle, *δ*(*ω*), are computed [25] and fitted with three different master curve models: sigmoidal [32], Christensen Anderson and Marasteanu (CAM) [33] and a new developed sigmoidal CAM model, respectively. The next section provides an overview of the different models used in this paper.

## 4. Master Curves Models

The first formulation (i.e., Model 1) selected for this investigation consists of the sigmoidal model first introduced by Pellinen and Witczak [32]:

(1) $$Log|E*(\omega )|=\delta +\frac{\alpha }{1+e^{\beta +\gamma \cdot Log\omega }}=\delta +\frac{\alpha }{1+e^{\beta +\gamma \cdot (Log\omega_{r}+Log\alpha_{T})}}$$

In Equation (1), |E*(*ω*)| is the dynamic modulus (GPa); *δ* is the minimum value of the dynamic modulus; *α* is value of the fitted dynamic modulus (*δ* +*α* = maximum dynamic modulus); *β* and *γ* are fitting parameters; *ω* is frequency (rad/s); *ω_r_* is the reduced frequency (rad/s), and *a_T_* is the shift factor based on the time–temperature superposition principle [33]. The corresponding phase angle, *δ*(*ω*), master curve can be obtained from the mathematical expression of |E*(*ω*)|, based on Booji and Thoone approximation [34] and on Kramers and Konig relationship [35]:

(2) $$\delta (\omega )≅\frac{\pi }{2}\cdot \frac{\partial \mathrm{Log}|E*(\omega )|}{\partial \mathrm{Log}(\omega )}=\frac{\pi }{2}\cdot \frac{\partial }{\partial \mathrm{Log}(\omega )}[\delta +\frac{\alpha }{1+e^{\beta +\gamma \cdot Log\omega }}]=-\frac{\pi }{2}\cdot \frac{\alpha \cdot \gamma \cdot e^{\beta +\gamma \cdot Log\omega }}{{\left(1+e^{\beta +\gamma \cdot Log\omega }\right)}^{2}}$$

The Christensen, Anderson and Marasteanu (CAM) model [33] is the second master curve expression (i.e., Model 2) adopted. The equations for |E*(*ω*)| and corresponding *δ*(*ω*) (derived base on the same logic of Equation (2) can be expressed as:

(3) $$|E(\omega )*|=E_{g}\cdot {\left[1+{\left(\frac{\omega }{t_{c}}\right)}^{v}\right]}^{-\frac{w}{v}}\Rightarrow \mathrm{Log}|E(\omega )*|={\mathrm{LogE}}_{g}-\frac{w}{v}\cdot \mathrm{Log}\left[1+{\left(10^{\mathrm{Log}\omega_{r}+\mathrm{Log}a_{T}-\mathrm{Log}t_{c}}\right)}^{v}\right]$$

(4) $$\delta (\omega )≅\frac{\pi }{2}\cdot \frac{\partial \mathrm{Log}|E(\omega )*|}{\partial \mathrm{Log}(\omega )}=-\frac{w\cdot {\left(10^{Log(\omega )-t_{c}}\right)}^{v}}{1+{\left(10^{Log(\omega )-t_{c}}\right)}^{v}}$$

In Equation (3), *E_g_* is the Glassy modulus of asphalt mixture ranges from 42 to 48 GPa [27,28,36,37], while *v*, *w*, and *t_c_* are fitting parameters. It needs to be mentioned that the CAM model [33] is an exponential based function and does not present any sigmoidal structure in its formulation. This exponential function tends to present a deviation in the function shape compared to an “S” shaped sigmoidal function. Therefore, the shape of both |E*(*ω*)| and *δ*(*ω*) master curves may be quite different compared to Model 1.

The third model (i.e., Model 3) consists of a combination of Models 1 and 2 and it is identified as the sigmoidal CAM model (SCM). This model was developed by combining the simplicity of the CAM model with the benefit of the sigmoidal function as can be seen in Equations (5) and (6).

(5) $$\begin{array}{l} \mathrm{Log}|E(\omega )*|={\mathrm{LogE}}_{g}-\frac{w}{v}\cdot \mathrm{Sigmoidal}\;\mathrm{function}\\ \Rightarrow \mathrm{Log}|E(\omega )*|=\mathrm{Log}E_{g}+\frac{w}{v}\cdot \frac{1}{{\left[1+e^{z\cdot \mathrm{Log}\omega +t_{c}}\right]}^{\frac{1}{v}}}=\mathrm{Log}E_{g}+\frac{w}{v}\cdot \frac{1}{{\left[1+e^{z\cdot \left(\mathrm{Log}\omega_{r}+\mathrm{Log}a_{T}\right)+t_{c}}\right]}^{\frac{1}{v}}} \end{array}$$

In Equation (5), *E_g_* is the Glassy modulus of asphalt mixture ranging from 42 to 48 GPa [37], while *v*, *w*, and *t_c_* are fitting parameters. Similar to Equations (2) and (4), the phase angle, *δ*(ω), can be expressed as:

(6) $$\delta (\omega )≅\frac{\pi }{2}\cdot \frac{\partial \mathrm{Log}|E(\omega )*|}{\partial \mathrm{Log}(\omega )}=-\frac{w\cdot z\cdot e^{z\cdot Log(\omega )+t_{c}}}{v^{2}\cdot {\left(1+e^{z\cdot Log(\omega )+t_{c}}\right)}^{1+\frac{1}{v}}}$$

## 5. Data Analysis

**Figure 3:**
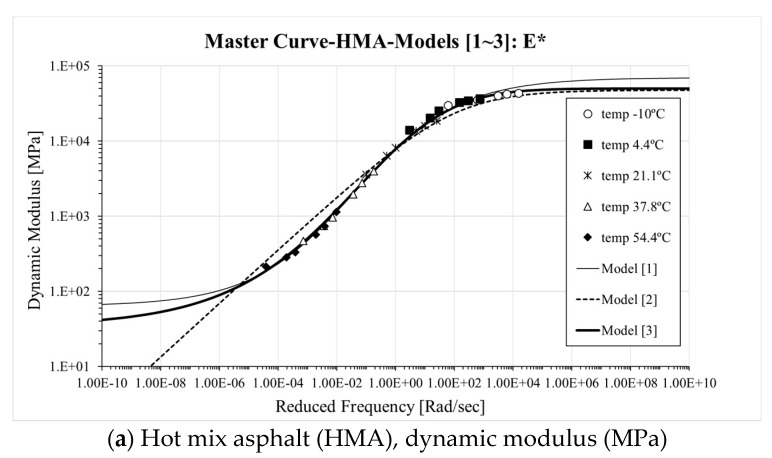

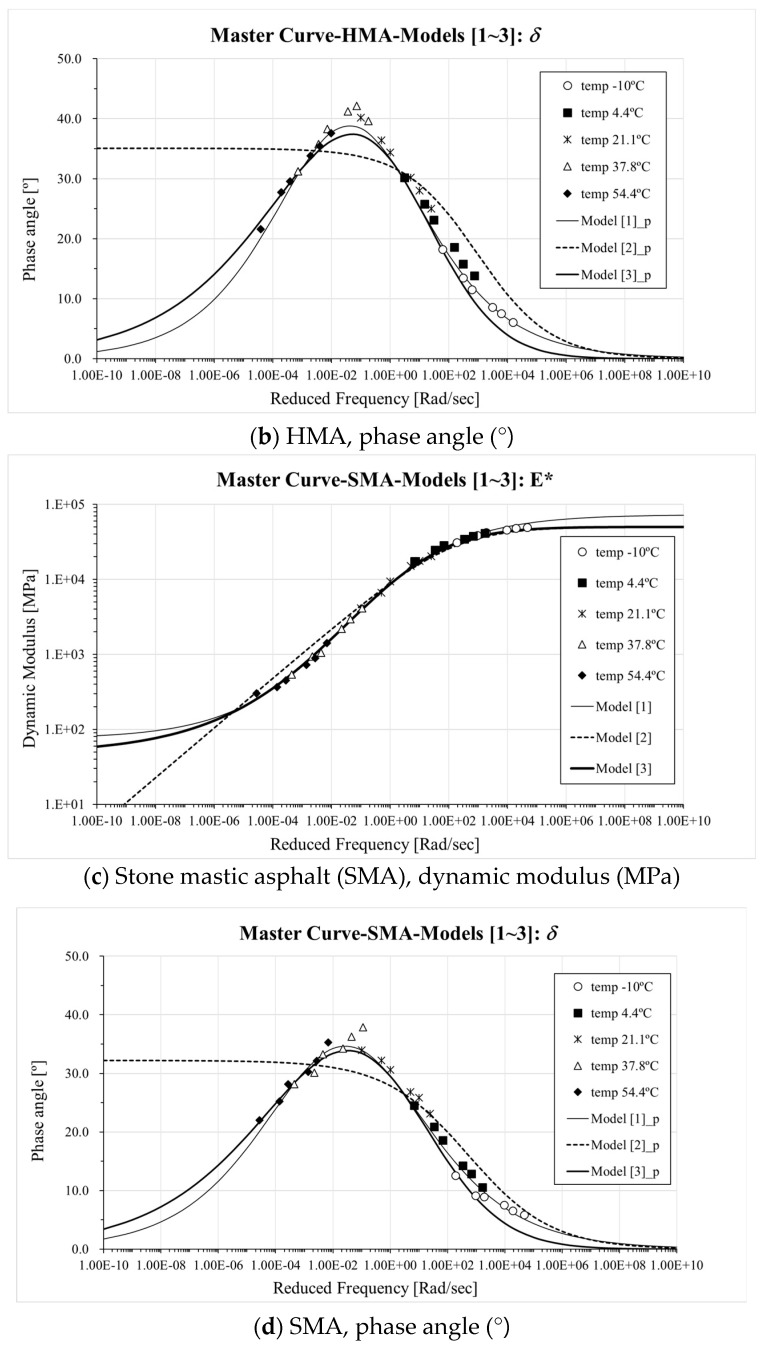

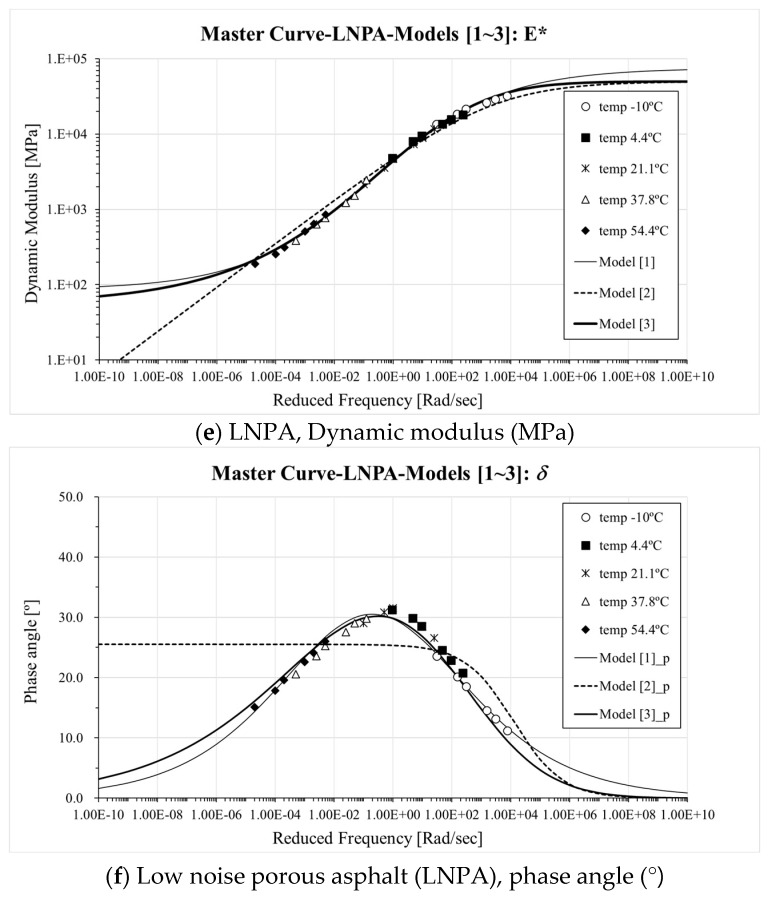
|E*(*ω*)| and *δ*(*ω*) master curves of Mixtures ((**a**,**b**) HMA, (**c**,**d**) SMA, (**e**,**f**) LNPA).

Both |E*(*ω*)| and *δ*(*ω*) expression are strictly [34,35] related for all three models; therefore, a simultaneous minimization approach can be performed to fit the experimental results as can be seen in Equation (7). The outcome of the fitting process is presented in Figure 3 and Table 3.

(7) $$\left\{ \begin{array}{l} Error_{\mathrm{for}\;\mathrm{Models}1,2,3}=\sum_{i=1}^{n}{\left[\mathrm{Experimental}\;-\;\mathrm{Model}\right]}^{2}\\ =\sum_{i=1}^{n}{\left[Log|E{\left(\omega \right)}_{e}*|-Log|E{\left(\omega \right)}_{f}*|\right]}^{2}(P\mathrm{art}\;[A])\\ +\sum_{i=1}^{n}{\left[Log\delta *{\left(\omega \right)}_{e}-Log\delta *{\left(\omega \right)}_{f}\right]}^{2}(P\mathrm{art}\;[B])≅0\;(\mathrm{Minimization}:\mathrm{Part}\;[A]+\mathrm{Part}\;[B]) \end{array}\right.$$

From the results in Figure 3, it can be observed that Model 1 (sigmoidal) and Model 3 (SCM: sigmoidal CAM Model) present a clear symmetric “S” shaped trend in case of predicting |E*(*ω*)| results over a wide range of frequencies. Model 1 shows higher |E*(*ω*)| both at low and high frequency compared to Model 3. In the case of Model 2 (CAM model), similar |E*(ω)| prediction compared to Model 3 is observed at high frequency. However, a different trend is exhibited at low frequency where a substantial deviations form models 1 and 3 are experienced.

In the case of *δ*(*ω*), the Booji and Thoone approximation [34] provides a reasonable level of fitting for Models 1 and 3. Nevertheless, the latter presents higher and lower values *δ*(*ω*) at low and high frequency compared to Model 1. On the other hand, poor predictions were found for the phase angle when using Model 2, especially at low frequency where a horizontal plateau is observed. Such a trend suggests that Model 3 (SCM model) can successfully provide upper and lower bounds for |E*(*ω*)| and *δ*(*ω*) which are relatively close to that obtained from the sigmoidal model. This is not the case when using the CAM model [33].

**Figure 4:**
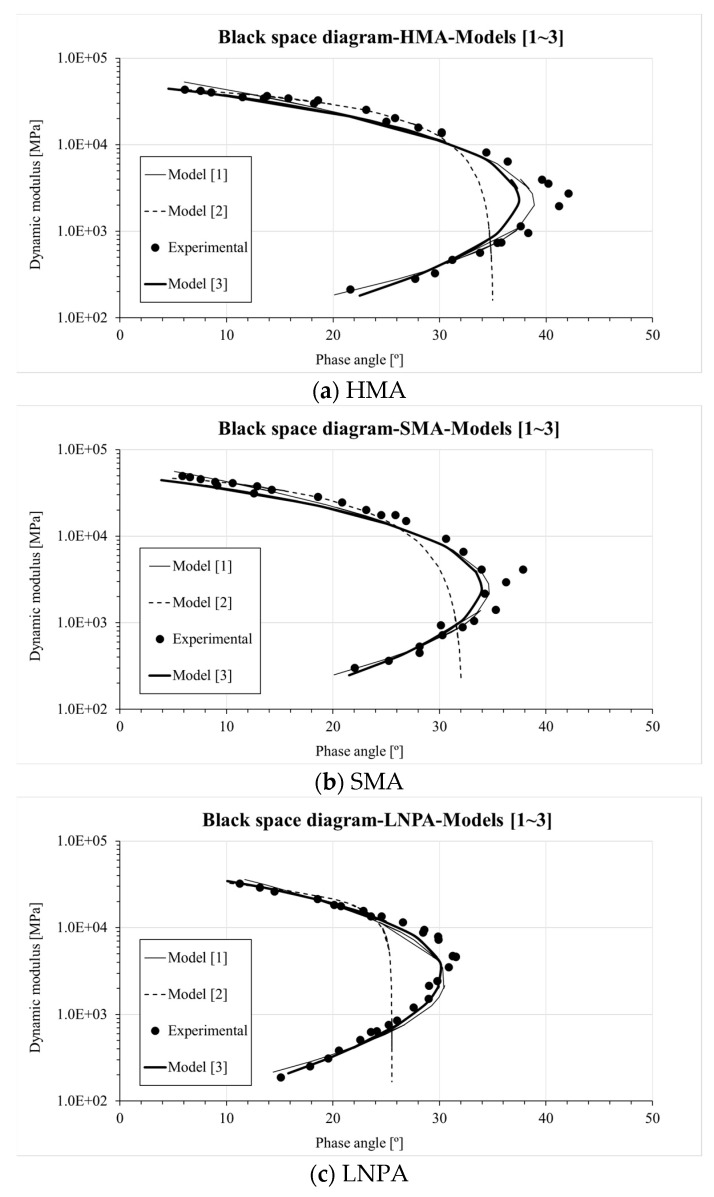
Black diagram-|E*(*ω*)| versus *δ*(*ω*)-for the tested asphalt mixtures ((**a**) HMA, (**b**) SMA, (**c**) LNPA).

In addition, the black space diagram was generated to further evaluate the difference between the three master curve formulations (Figure 4). Similarly to Figure 3, comparable fitting can be observed for Models 1 and 3. This also supports the application of the Booji and Thoone approximation [34] together with the fitting approach adopted in Equation (7). However, a considerably distinct prediction trend was found when using the CAM model (Model 2) [33]; this largely deviates from the experimental measurements.

A simple *t*-test (i.e., hypothesis test) with a 5% significance level was adopted to statistically estimate the difference in the fitting capability of the three master curves models. The range of *t*-test comparisons was from 1^−10^ to 1^10^ rad/s with exponential scale (base 10) intervals. Assumptions of data normality and constant variance were imposed and the statistical testing hypotheses were set as [38]:

(8) $$\mathrm{Null}\;\mathrm{hypothesis}:\;H_{0}:\mu_{A\_Group:E*or\delta }=\mu_{B\_Group:E*or\delta }$$

(9) $$\mathrm{Alternative}\;\mathrm{hypothesis}:\;H_{0}:\mu_{A\_Group:E*or\delta }\neq \mu_{B\_Group:E*or\delta }$$

In Equations (8) and (9), the mean value, *μ*, corresponds to |E*(*ω*)| (and/or *δ* (*ω*)) obtained by fitting each master curve model to the experimental data. In addition, the pooled standard deviation, *S_P_*, can be computed as follows:

(10) $$S_{P[E*or\delta ]}=\sqrt{\frac{(n_{A}(=3)-1)\cdot S_{A,[E*or\delta ]}^{2}+(n_{B}(=3)-1)\cdot S_{B[E*or\delta ]}^{2}}{n_{A}(=3)+n_{B}(=3)-2=4}}$$

In Equation (10), $S_{A[E*or\delta ]}$ is the standard deviation of |E*(*ω*)| (and/or *δ*(*ω*)) (Group A); $S_{B[E*or\delta ]}$ is the standard deviation of |E*(*ω*)| (and/or *δ*(*ω*)) (Group B); *n*_A_ and *n*_B_ are the numbers of tested specimens in Group A and B (*n* = 3). Then, the results of *t*-static can be computed from Equation (10) as:

(11) $$t-\mathrm{static}=\frac{\mu_{A[E*or\delta ]}-\mu_{B[E*or\delta ]}}{S_{P[E*or\delta ]}\cdot \sqrt{\frac{1}{n_{A}}+\frac{1}{n_{B}}=\frac{1}{3}+\frac{1}{3}}}=\frac{\mu_{A[E*or\delta ]}-\mu_{B[E*or\delta ]}}{S_{P[E*or\delta ]}\cdot \sqrt{\frac{2}{3}}}$$

Finally, the output of this statistical test, the *p*-value, can be computed based on Equations (8)–(11). If the *p*-value is less than 0.05 (i.e., significance level), the two compared groups are statistically different, otherwise, they can be assumed as statistically equivalent. All the computed results are presented in Table 3 and Table 4.

Distinct differences in |E*(*ω*)| can be observed between Model 1 (sigmoidal) and Model 2 (CAM model) at almost all tested frequencies. In addition, Model 1 (sigmoidal) and Model 3 (SCM model) result in significantly different predictions at low and high frequencies confirming the overall impression obtained from a simple visual inspection of the master curves. Within these two models, similar fitting of |E*(ω)| is found for a mid-frequency range (i.e., between 10^−4^ and 10^2^ rad/s). When comparing Model 2 (CAM model) and Model 3 (SCM model), statistically distinct values |E*(*ω*)| at low and intermediate frequency are found while the model return equivalent predictions at high frequency. In the case of *δ* (*ω*), except for the mid-frequency range (i.e., 10^−2^ to 10^2^ rad/s) Model 1 and Model 3 present distinct values. In the case of the CAM model, the statistical analysis confirms that the large deviation of the model prediction from the experimental measurements. Based on the results of the analysis performed, SCM can be potentially used as an alternative mathematical model to the conventional sigmoidal function for providing upper (and/or lower) limits for |E*(*ω*)| and *δ*(*ω*). This is because Model 3 (SCM) presents the characteristics not only of a sigmoidal formulation but also the benefits of the simple expression of typical of the CAM model.

## 6. Composite Pavement Performance Simulations

A commercial pavement performance analysis program named: AASHTOWare-Pavement (mechanistic-empirical pavement design guide—MEPDG) is used in the present research effort to evaluate the effect of incorporating the three different rheological master curve models on the performance prediction of a composite pavement system [30]. It needs to be mentioned that overlay pavement analysis (e.g., asphalt layer on existing concrete pavement layer) started to become available from the present commercial version of MEDPG (version 2.5.3). In MEPDG analysis the following steps are needed to analyze pavement performance:

(1) Input pavement analysis type (e.g., newly pavement or overlay; asphalt, concrete or composite pavement layer) and expected service life along with pavement performance criteria (e.g., smoothness, cracking resistance, rutting resistance, etc.)
(2) Input traffic, climate, pavement layer bondage effects
(3) Input material properties of asphalt, concrete pavement layer along with cemented base layer (if exists). In this step, dynamic modulus of asphalt pavement layer with three different models is used as crucial input parameter (e.g., MEPDG input parameter Level 1)
(4) Perform analysis and evaluate the computed results

**Figure 5:**
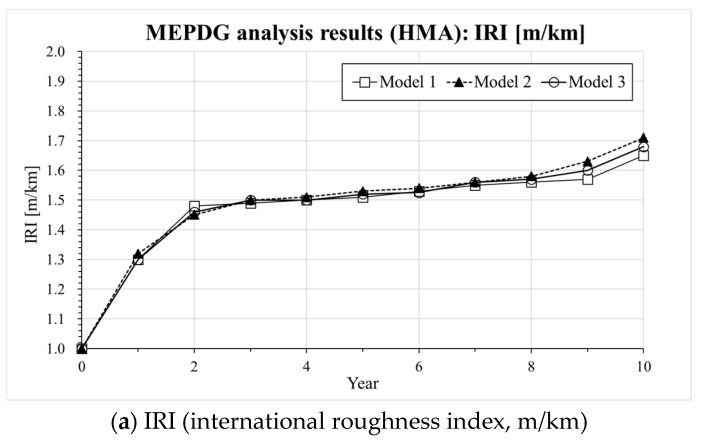

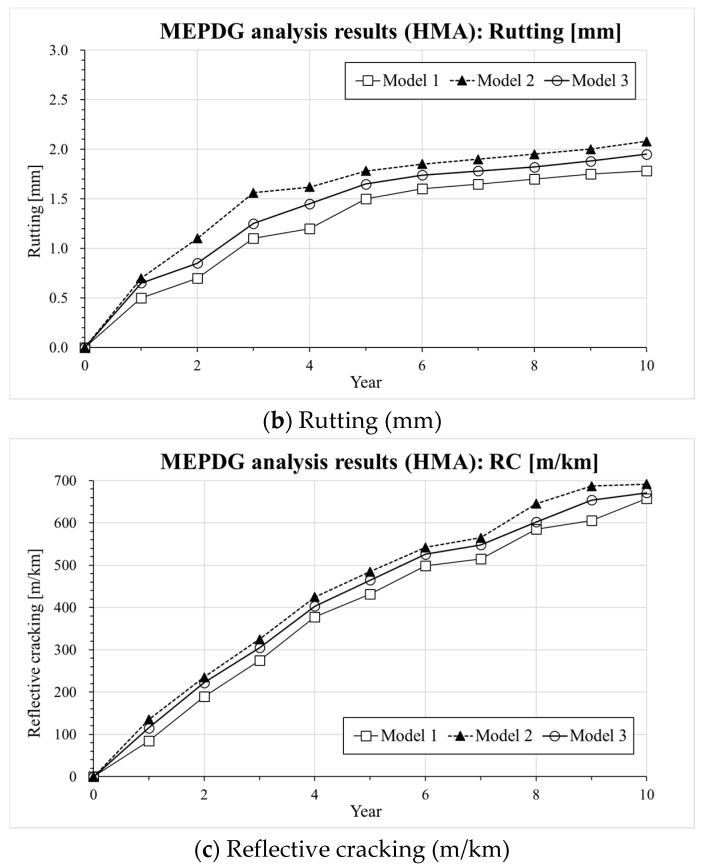
Results of AASHTOWARE analysis for Mixture 1 (HMA): (**a**) IRI, (**b**) Rutting, (**c**) Reflective cracking.

**Figure 6:**
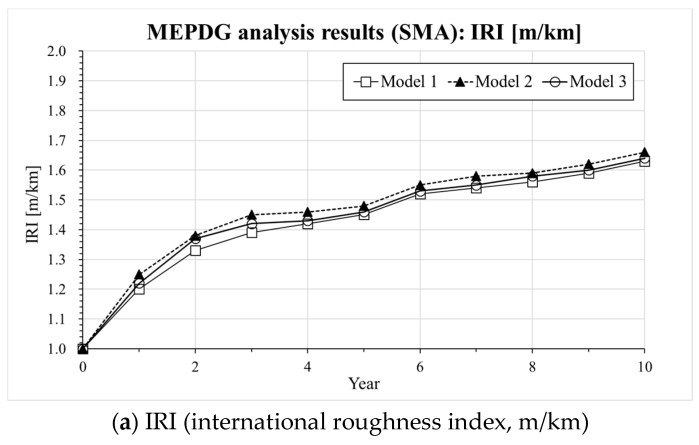

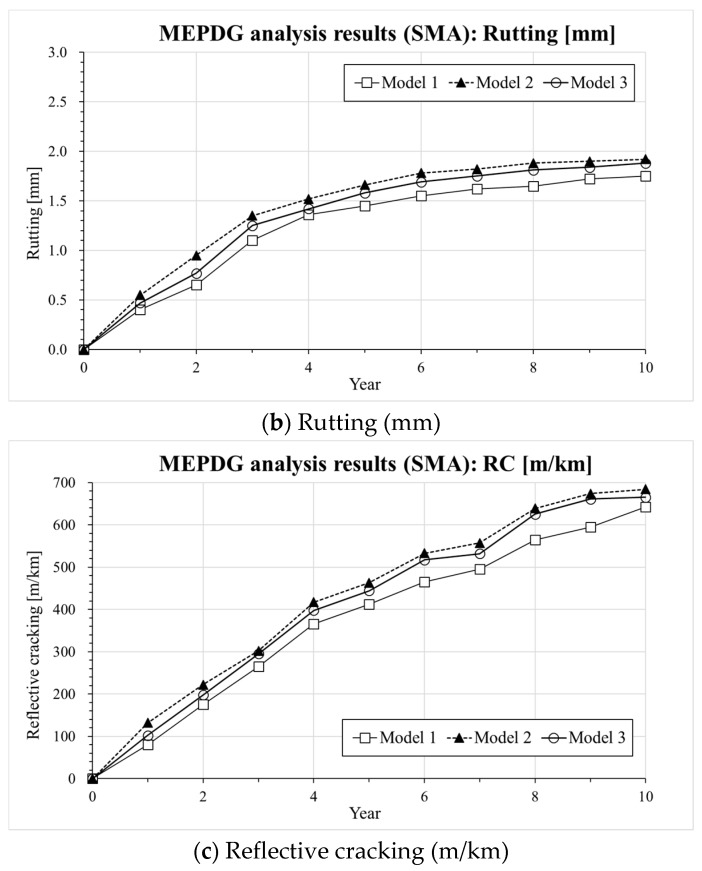
Results of AASHTOWARE analysis for Mixture 2 (SMA): (**a**) IRI, (**b**) Rutting, (**c**) Reflective cracking.

**Figure 7:**
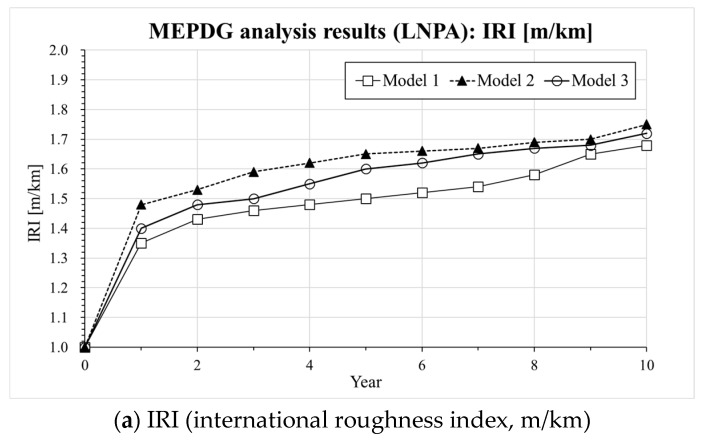

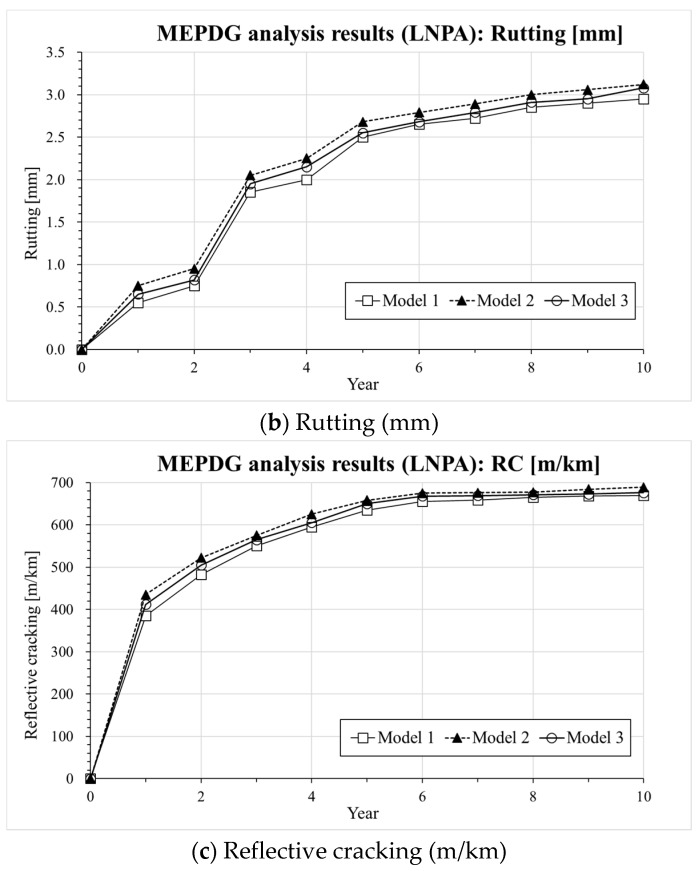
Results of AASHTOWARE analysis for Mixture 3 (LNPA): (**a**) IRI, (**b**) Rutting, (**c**) Reflective cracking.

Information on the input parameters and computation assumptions adopted for the MEPDG analysis [30] is shown in Table 5. It should be noticed that the thickness of the pavement layer and the design parameters are set based on the current expressway design guide (e.g., composite pavement design guide written in Korea Expressway Corporation) in South Korea [29]. In the case of LNPA (Mixture 3), only an upper layer with 5 cm thickness is considered while the lower 5 cm asphalt mixture layer consisted of an SMA mixture based on the current composite LNPA design guide in South Korea [29]. Figure 5, Figure 6 and Figure 7 present the results of the MEPDG analysis in terms of IRI, rutting and Reflective Cracking when the three master curves models investigated in the present study are incorporated in the AASHTOWare-Pavement simulation. 

The results presented in Figure 5, Figure 6 and Figure 7 indicate that there is a similar trend in the distress estimation (e.g., IRI, rutting and reflective cracking) for all three different |E*(ω)| master curve models. In all cases, Model 2 (CAM model [33]) presents the highest values compared to Model 3 (SCM model) and Model 1 (sigmoidal model) which show the intermediate and lowest level of distress estimation, respectively. The differences in IRI prediction are not that significant among the three models except for the LNPA case, for which the presence of a high degree of air voids might ultimately affect the evolution of this parameter. However, distinct upper and lower bounds on rutting and reflective cracking are obtained for all tested cases. In the previous section, a higher |E*(ω)| could be predicted with Model 1 (sigmoidal) compared to Model 3 (SCM model). This reflects in the actual response of the pavement predicted with the AASHTOWare software. It also needs to be mentioned that the results of the MEPDG analysis performed with Model 2 (CAM model [33]) might be not necessarily very robust as a poor prediction of dynamic modulus and phase angle at a higher temperature and low frequency was observed (deviation from the experimental data). In view of the plots presented in Figure 5, Figure 6 and Figure 7, while the MEPDG output of Model 1 (sigmoidal) and Model 3 (SCM) appears to be relatively close, the latter provides a more conservative prediction of the evolution of the pavement distresses (higher degree of distress). Such preliminary results may suggest the opportunity of incorporating the SCM model in the design software to guarantee a potentially more reliable approach to the pavement design procedure eventually leading to longer lasting road infrastructures.

## 7. Summary and Conclusions

In this paper, three different mathematical models, including a newly developed model (sigmoidal CAM model (SCM)), are considered to generate dynamic modulus (and corresponding phase angle) master curves and next evaluated in the pavement design of rehabilitated concrete pavements subjected to asphalt overlay, based on the mechanistic-empirical pavement design guide (MEPDG) procedure performed with a commercially available software, AASHTOWare. As a result, the SCM model could successfully provide lower limits when the fitting of experimental data compared to the conventionally adopted sigmoidal model. The pavement design analysis showed similar results in the prediction of the various pavement distress levels for composite pavement structure, such as IRI, rutting and reflective cracking. While the pavement response obtained with the CAM model appears to be not sufficiently reliable especially in the high temperature domain, the pavement design analysis performed with SCM seems to provide a higher degree of distresses compared to the results obtained with the conventional sigmoidal function. This results in a more conservative evaluation of the pavement and of its performance during its service life. While this may eventually lead to the design of more durable pavement with higher service quality when an overlay is adopted, an economic analysis would need to be performed to obtain an overall estimation of the actual benefit of using a different master curve model in the pavement design procedure. In addition, the possibility of developing a series of recommendations for design purposes could be considered after a wider set of data possibly including field response is obtained. However, it must be mentioned that only three mixture types are considered in this paper for performing the overlay treatment. Additional materials and a wider range of mathematical models for dynamic modulus master curves should be evaluated in a follow-up research effort to further verify the findings obtained in the present investigation.

## Figures and Tables

**Table 1:** Mix design of the asphalt mixtures.

Mix ID	Mixture Description (Mixture Type)	Detailed Mixture Information (Aggregate Passing Sieve %)
HMA (Mixture 1)	Hot Mix Asphalt (NMAS = 13 mm) (WC-1)	Air Voids = 4.3–4.5%/OAC = 4.4–4.6% VMA = 14.3–14.8%/VFA = 71–73% (Granite: 13 mm: 92%, 5 mm: 56%, 2.5 mm: 39%, 0.6 mm: 25%, 0.3 mm: 15%, 0.08 mm: 6%)
SMA (Mixture 2)	Stone Mastic Asphalt (NMAS = 13 mm) (SMA-13)	Air Voids = 2.2–2.5%/OAC = 6.3–6.6% VMA = 17.2–17.9%/VFA = 78.3–79.8% (Granite: 13 mm: 100%, 10 mm: 48%, 2.5 mm: 15%, 0.6 mm: 12%, 0.3 mm: 9%, 0.08 mm: 7%)
LNPA (Mixture 3)	Low Noise Porous Asphalt (NMAS = 10 mm) (LNPA-10)	Air Voids = 18–22%/OAC = 6.3–6.8% VMA = 31.2–31.7%/VFA = 38.9–44.2% [Granite: 13 mm: 100%, 10 mm: 90%, 5 mm: 31%, 0.6 mm: 10%, 0.3 mm: 7%, 0.08 mm: 5%]

OAC: optimum asphalt content; VMA: voids in the mineral aggregates; VFA: voids Filled with asphalt; NMAS: nominal maximum aggregate size.

**Table 2:** Dynamic modulus-testing information.

Contents	Dynamic Modulus (DM) Testing Conditions
Specimen size	Diameter: 100 mm; Specimen height: 150 mm; Gauge length: 100 mm
# of replicates	Three specimens per mixture type (total of nine specimens)
Temperature	−10, 4.4, 21.1, 37.8 and 54.4 °C (21.1 °C was set as reference temperature: *T*_S_)
Frequency	0.1, 0.5, 1, 5, 10 and 25 rad/s
Others	Three LVDT sensors are used (LVDT: Linear Variable Displacement Transducer)

**Table 3:** Results of *t*-test for |E*(*ω*)| comparison.

Mixture Type	Angular Frequency (rad/s)	*p*-Value (Significance Level: α = 0.05) [N]: Non-Significant, [S]: Significant		
		Model 1 Vs. Model 2	Model 1 Vs. Model 3	Model 2 Vs. Model 3
HMA (Mix 1)	10^−10^	0.0000 [Sig]	0.0000 [Sig]	0.0000 [Sig]
	10^−8^	0.0001 [Sig]	0.0003 [Sig]	0.0001 [Sig]
	10^−6^	0.0015 [Sig]	0.0011 [Sig]	0.0502 [Non]
	10^−4^	0.0089 [Sig]	0.0912 [Non]	0.0075 [Sig]
	10^−2^	0.0092 [Sig]	0.1231 [Non]	0.0065 [Sig]
	10	0.0412 [Sig]	0.1156 [Non]	0.0252 [Sig]
	10^2^	0.0504 [Non]	0.0654 [Non]	0.0375 [Sig]
	10^4^	0.0075 [Sig]	0.0098 [Sig]	0.0482 [Sig]
	10^6^	0.0023 [Sig]	0.0082 [Sig]	0.0512 [Non]
	10^8^	0.0005 [Sig]	0.0023 [Sig]	0.0985 [Non]
	10^10^	0.0001 [Sig]	0.0005 [Sig]	0.1123 [Non]
SMA (Mix 2)	10^−10^	0.0000 [Sig]	0.0002 [Sig]	0.0000 [Sig]
	10^−8^	0.0001 [Sig]	0.0004 [Sig]	0.0000 [Sig]
	10^−6^	0.0004 [Sig]	0.0013 [Sig]	0.0002 [Sig]
	10^−4^	0.0072 [Sig]	0.1212 [Non]	0.0058 [Sig]
	10^−2^	0.0101 [Sig]	0.1542 [Non]	0.0061 [Sig]
	10	0.0455 [Sig]	0.2565 [Non]	0.0142 [Sig]
	10^2^	0.0515 [Non]	0.0512 [Non]	0.0485 [Sig]
	10^4^	0.0154 [Sig]	0.0075 [Sig]	0.0512 [Non]
	10^6^	0.0012 [Sig]	0.0062 [Sig]	0.0684 [Non]
	10^8^	0.0006 [Sig]	0.0025 [Sig]	0.0845 [Non]
	10^10^	0.0001 [Sig]	0.0004 [Sig]	0.1245 [Non]
LNPA (Mix 3)	10^−10^	0.0000 [Sig]	0.0003 [Sig]	0.0001 [Sig]
	10^−8^	0.0001 [Sig]	0.0011 [Sig]	0.0001 [Sig]
	10^−6^	0.0002 [Sig]	0.0085 [Sig]	0.0007 [Sig]
	10^−4^	0.0041 [Sig]	0.1845 [Non]	0.0021 [Sig]
	10^−2^	0.0085 [Sig]	0.2545 [Non]	0.0031 [Sig]
	10	0.0174 [Sig]	0.3241 [Non]	0.0285 [Sig]
	10^2^	0.0211 [Sig]	0.0845 [Non]	0.0185 [Sig]
	10^4^	0.0111 [Sig]	0.0745 [Non]	0.0345 [Sig]
	10^6^	0.0011 [Sig]	0.0041 [Sig]	0.0511 [Non]
	10^8^	0.0003 [Sig]	0.0011 [Sig]	0.1422 [Non]
	10^10^	0.0001 [Sig]	0.0002 [Sig]	0.1674 [Non]

*Sig.: Statistically different, Non: Statistically not significant

**Table 4:** Results of *t*-test for *δ*(*ω*) comparison.

Mixture Type	Angular Frequency (rad/s)	*p*-Value (Significance Level: α = 0.05) [N]: Non-Significant, [S]: Significant		
		Model 1 Vs. Model 2	Model 1 Vs. Model 3	Model 2 Vs. Model 3
HMA (Mix 1)	10^−10^	0.0004 [Sig]	0.0006 [Sig]	0.0001 [Sig]
	10^−8^	0.0008 [Sig]	0.0011 [Sig]	0.0001 [Sig]
	10^−6^	0.0001 [Sig]	0.0001 [Sig]	0.0003 [Sig]
	10^−4^	0.0021 [Sig]	0.0232 [Sig]	0.0009 [Sig]
	10^−2^	0.0121 [Sig]	0.0092 [Sig]	0.0012 [Sig]
	10	0.0545 [Non]	0.0022 [Sig]	0.0023 [Sig]
	10^2^	0.0135 [Sig]	0.0435 [Sig]	0.0135 [Sig]
	10^4^	0.0025 [Sig]	0.0055 [Sig]	0.0025 [Sig]
	10^6^	0.0825 [Non]	0.0125 [Sig]	0.0012 [Sig]
	10^8^	0.0644 [Non]	0.0214 [Sig]	0.0512 [Non]
	10^10^	0.1221 [Non]	0.1221 [Non]	0.0616 [Non]
SMA (Mix 2)	10^−10^	0.0001 [Sig]	0.0006 [Sig]	0.0001 [Sig]
	10^−8^	0.0001 [Sig]	0.0007 [Sig]	0.0001 [Sig]
	10^−6^	0.0001 [Sig]	0.0011 [Sig]	0.0001 [Sig]
	10^−4^	0.0001 [Sig]	0.0085 [Sig]	0.0011 [Sig]
	10^−2^	0.0085 [Sig]	0.0512 [Non]	0.0021 [Sig]
	10	0.0548 [Non]	0.0611 [Non]	0.0008 [Sig]
	10^2^	0.0085 [Sig]	0.0411 [Sig]	0.0081 [Sig]
	10^4^	0.0002 [Sig]	0.0041 [Sig]	0.0015 [Sig]
	10^6^	0.0745 [Non]	0.0032 [Sig]	0.0011 [Sig]
	10^8^	0.0822 [Non]	0.0481 [Sig]	0.0544 [Non]
	10^10^	0.1541 [Non]	0.1154 [Non]	0.0584 [Non]
LNPA (Mix 3)	10^−10^	0.0001 [Sig]	0.0005 [Sig]	0.0001 [Sig]
	10^−8^	0.0001 [Sig]	0.0008 [Sig]	0.0001 [Sig]
	10^−6^	0.0001 [Sig]	0.0010 [Sig]	0.0001 [Sig]
	10^−4^	0.0001 [Sig]	0.0052 [Sig]	0.0008 [Sig]
	10^−2^	0.0002 [Sig]	0.0541 [Non]	0.0010 [Sig]
	10	0.0005 [Sig]	0.0712 [Non]	0.0015 [Sig]
	10^2^	0.0085 [Sig]	0.0845 [Non]	0.0041 [Sig]
	10^4^	0.0002 [Sig]	0.0021 [Sig]	0.0011 [Sig]
	10^6^	0.0633 [Non]	0.0011 [Sig]	0.0544 [Non]
	10^8^	0.0912 [Non]	0.0312 [Sig]	0.0845 [Non]
	10^10^	0.1412 [Non]	0.0412 [Sig]	0.0984 [Non]

**Table 5:** Input parameters for the AASHTOWare mechanistic-empirical pavement design guide (MEPDG) analysis.

Contents	Descriptions	Pavement Structure
Considered Expressway	Ho-Nam expressway (South Korea) AADT: 31,043 (More than class 3: 6,852) -Two lanes in design direction Traffic growth rate: 1.15–7.03% -Compound increase equation was applied Climate: Jeon-Nam location (relatively hot and humid during summer and cold during winter)	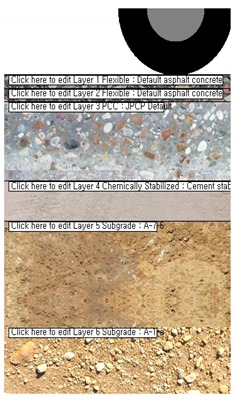 Composite pavement
Asphalt pavement (*t* = 10 cm)	Asphalt binder: PG 76-22/PG 82-34, Level 1 Creep compliance: Level 3(Aggregate gradation) Dynamic modulus: Level 1 (used estimated values with Models 1–3) -Other properties were input based on Table 1	
Concrete Pavement (*t* = 30 cm)	Compressive strength = 34 MPa (Level 2) -*ν* = 0.21, *ρ* = 2480 kgf/m^3^, W/C = 0.42 -LTE = 55%, Dowel = D (32 mm)/350 mm (LTE: Load Transfer Efficiency: 0–100%) -Tie bar: Installed, Widened-slab: Installed, 0.5 m -Joints are placed every 6 m Aggregate = Granite, Cement = Type 1(Ordinary Portland Cement)	
Lean Concrete (*t* = 15 cm)	Elastic/resilient modulus = 13,800 MPa -*ν* = 0.20, *ρ* = 2390 kgf/m^3^	
Sub grade (infinite)	AASHTO A-7-6 AASHTO A-1-A	
Distress level: limits in South Korea	IRI = 3.0 m/km, AC Bottom-up cracking = 25% AC Rutting/thermal cracking = 13 mm/378 m/km AC Reflective cracking = 684 m/km Pavement performance evaluation for 10 years	
